# Simulated increased soft tissue thickness artefactually decreases trabecular bone score: a phantom study

**DOI:** 10.1186/s12891-016-0886-1

**Published:** 2016-01-13

**Authors:** Sasithorn Amnuaywattakorn, Chanika Sritara, Chirawat Utamakul, Wichana Chamroonrat, Arpakorn Kositwattanarerk, Kanungnij Thamnirat, Boonsong Ongphiphadhanakul

**Affiliations:** Department of Diagnostic and Therapeutic Radiology, Faculty of Medicine Ramathibodi Hospital, Mahidol University, Bangkok, 10400 Thailand; Department of Medicine, Faculty of Medicine Ramathibodi Hospital, Mahidol University, Bangkok, 10400 Thailand

**Keywords:** Body mass index, Bone mineral density, Dual X-ray absorptiometry, Phantom, Least significant change, Precision, Trabecular bone score

## Abstract

**Background:**

Trabecular bone score (TBS), which has been proposed to be used in complementary with bone mineral density (BMD) to improve the assessment of fracture risk, is negatively associated with body mass index (BMI). The effect of soft tissue, which is expected to be thicker in subjects with high BMI, on TBS was studied using three scan types: Hologic with fast array mode (Hfa), Hologic with high definition mode (Hhd), and GE-Lunar iDXA.

**Methods:**

A spine phantom provided by Hologic for routine quality control procedure was scanned using three scan types: Hfa, Hhd, and iDXA. The phantom was scanned with an overlying soft tissue equivalent material (bolus used in radiotherapy) of 0 (without), 1, 2.5, 3.5, 5 and 7.5 cm thick. For each setting, 30 acquisitions were performed in the same way as for the quality control procedure. TBS was calculated using TBS iNsight® software version 2.1 on the same regions of interest as those used for lumbar spine BMD.

**Results:**

Mean ± SD TBS of the phantom (without overlying soft tissue) were 1.379 ± 0.018, 1.430 ± 0.009, and 1.423 ± 0.005 using Hfa, Hhd, and iDXA, respectively. A one-way repeated measures ANOVA showed that there were statistically differences in TBS due to different thicknesses of soft tissue equivalent materials for all three scan types (*p* < 0.001). A Tukey post-hoc test revealed that the decrease in TBS was statistically significant (*p* < 0.001) when the soft tissue thickness was 1 cm (−0.0246 ± 0.0044, −0.0319 ± 0.0036, and −0.0552 ± 0.0015 for Hfa, Hhd, and iDXA, respectively). Although to a lesser degree, the effects were also statistically significant for BMD (*p* < 0.05): an increase for Hfa and Hhd but a decrease for iDXA. However, these changes did not exceed the least significant change (LSC) derived from patients.

**Conclusions:**

Increased soft tissue thickness results in lower TBS value. Although BMD is also affected, it is unlikely to pose a clinical problem because the change is unlikely to exceed the patient-derived LSC.

## Background

Osteoporosis leading to fractures and increased morbidity and mortality in the elderly has been a health problem worldwide. The standard screening procedure is bone mineral density (BMD) assessment using dual X-ray absorptiometry (DXA). Although BMD identifies many individuals at risk of fracture, a large degree of overlap exists in BMD values between individuals who develop fractures and those who do not [1]. Since BMD does not capture all of the aspect of fracture risk, trabecular bone score (TBS) has been proposed to improve the assessment [2]. TBS is a texture parameter postulated to reflect bone microarchitecture, i.e., the number of connections among bone trabeculae [3]. It is derived from a lumbar spine image acquired by dual X-ray absorptiometry for the assessment of BMD. Using software to analyze the very same lumbar spine region of interest (ROI), the variation among the image pixels is assessed [4]. High variation among the pixels results in high TBS value. Low TBS has been found to be associated with fractures [3]. Ex vivo studies has revealed significant correlation with connectivity of bone trabeculae [5] and trabecular bone volume as well as compressive stiffness [6]. Its ability to discriminate between women with and without fractures has been documented [7].

A negative association between TBS and body mass index (BMI) has been reported [8], which is compatible with the finding in our cross-sectional study to derive age-adjusted reference data [9], suggesting weaker bones in individuals with higher BMI. This view is controversial. Although it is supported by a study in premenopausal women with various BMI using trans-iliac bone biopsies, which showed that bones of obese subjects were of poorer quality with a lower number of trabecular connections [10], more recent studies showed the opposite findings with significantly greater trabecular number by high-resolution peripheral quantitative computed tomography and higher bone strength by micro-finite element analysis [11–13].

However, subjects with high BMI are likely to have thicker soft tissue, which could act like a blurring filter to diminish clarity of the DXA image. The variations among the image pixels could be more difficult to detect, resulting in underestimation of TBS. To study the effect of increased BMI or increased soft tissue thickness, the bone structure should be constant while the thickness of the soft tissue varies. Therefore, a study on a bone phantom serves the purpose.

Since there is no specific phantom provided for TBS, researchers have attempted TBS analysis on BMD quality control phantoms provided by the DXA manufacturers. TBS analysis on a Hologic BMD quality control phantom showed a TBS image resembling the variations seen in vivo [14]. Using the phantom to avoid patients’ exposure to radiation, Bandirali et al. [15] conducted a reproducibility study of TBS on a Hologic DXA system and concluded that the three Hologic scan modes, i.e., fast array, array, and high definition, could be used interchangeably despite the differences being statistically significant because they were within the highest least significant change (LSC). Since the array mode took longer time (75 seconds) than the fast array mode (40 seconds), the latter was used in our routine clinical practice. However, we were interested in comparing it with the high definition mode, which was supposed to give the best image quality despite the fact that it took the longest time (145 seconds). Because we also used an iDXA system (GE/Lunar, Madison, WI) in our routine work, it was of our interest to explore the effect of soft tissue on the derived TBS. However, the Hologic phantom was also used with the iDXA system because the TBS image of the iDXA phantom did not resemble that of a human lumbar spine [14]. For iDXA, the acquisition mode is automatically chosen by the system according to the patient’s BMI.

We aimed at investigating the effect of soft tissue thickness on TBS assessment using three scan types, i.e. Hologic with fast array mode (Hfa), Hologic with high definition mode (Hhd) (Hologic Discovery A, Bedford, MA), and iDXA. For the sake of comparison, the effect on BMD was also studied.

## Methods

**Fig. 1:**
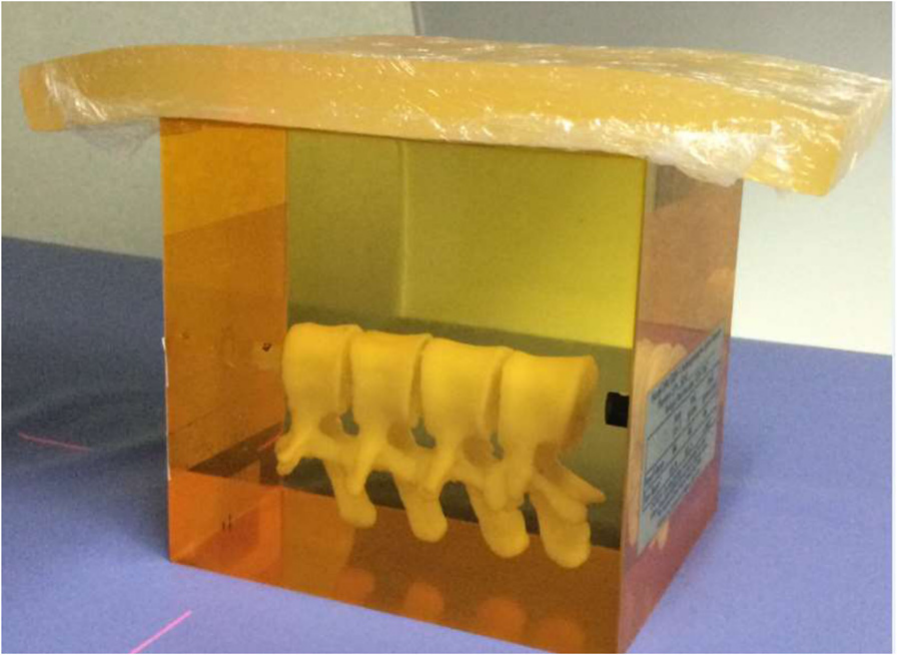
Lumbar spine phantom setup. A Hologic BMD spine phantom, for routine quality control of BMD assessment, with a soft tissue equivalent material placed on top

A spine phantom provided by Hologic for routine quality control procedure was scanned using three scan types: Hfa, Hhd, and iDXA. The phantom was scanned alone and with an overlying, commercially available, soft tissue equivalent material, the so-called “bolus” (Superflab Bolus Material™, Nuclear Associates, NY, USA) of 1, 2.5, 3.5, 5 and 7.5 cm thickness (Fig. 1). The bolus was used because it has been shown to be comparable to soft tissue in terms of number of electrons per gram, effective atomic number, density, and radiation attenuation coefficient [16–18]. It has long been used in radiotherapy applications, one of which was to compensate for uneven or missing tissue [19]. For each setting, 10 acquisitions were performed in the same way as the quality control procedure (60 acquisitions for each scan type, 180 acquisitions altogether). Using TBS iNsight® software version 2.1 (Medimaps, Geneva, Switzerland) on the same regions of interest (ROI) as those used for lumbar spine BMD, TBS was calculated. For analysis of the first acquisition, the ROI was automatically generated by the system and adjusted by the technologist as necessary. Once the ROI was considered appropriate, it was copied for reuse on subsequent acquisitions.

For Hfa, the BMD and TBS LSC values derived from volunteers were 1.91 and 5.68 %, respectively [9]. The BMD LSC derived from representative patients was 1.19 % for iDXA. There was no previously established LSC for Hhd because it was not routinely used in our routine practice.

### Statistical analysis

To check the assumptions required for one-way repeated measures ANOVA, TBS and BMD data were assessed for outliers (i.e., the values larger than three times the distance between the median and the lower/upper quartile) using box-and-whisker plots. Normality of the data were examined using Shapiro-Wilk test. Mean ± SD and 95 % confidence interval (95 % CI) of BMD and TBS with various thicknesses of soft tissue equivalent material were calculated and the means of TBS were compared using one-way repeated measures ANOVA with and without Greenhouse-Geisser correction for sphericity. Then Tukey post-hoc test was performed to determine which thickness of soft tissue equivalent material differed from each other. *P* value of < 0.05 was considered statistically significant. The values of LSC of TBS and BMD of each of the three types of scans were calculated as 2.77 x the coefficient of variation (%) [20] and its complement to 100 % was used as a measure of reproducibility [15]. All calculations were performed using Stata 12 software (StataCorp, College Station, TX).

## Results

Mean ± SD TBS of the phantom (without overlying soft tissue) were 1.379 ± 0.018, 1.430 ± 0.009, and 1.423 ± 0.005 using Hfa, Hhd, and iDXA, respectively (Table 1). The respective BMD values were 1.006 ± 0.004, 1.002 ± 0.004, and 1.139 ± 0.001 (Table 2). Among the three scan modes, the differences in mean TBS and mean BMD of the phantom itself (soft tissue 0 cm) were statistically significant: F(2, 58) = 150.75, *p* < 0.001; F(2, 58) = 19,642.61, *p* <0.001, respectively. The LSC values of the phantom TBS and BMD were shown in Table 3.

**Table 1:** TBS of a Lumbar Spine Phantom with Various Thicknesses of Soft Tissue Equivalent Material

Soft	Hologic Fast Array (Hfa)				Hologic HD (Hhd)				iDXA			
Tissue			95%CI				95%CI				95 % CI	
(cm)	mean	SD	Lower	Upper	mean	SD	Lower	Upper	mean	SD	Lower	Upper
0	1.379	0.018	1.372	1.386	1.430	0.009	1.427	1.433	1.423	0.005	1.421	1.424
1	1.355*	0.018	1.348	1.361	1.398*	0.008	1.395	1.401	1.367*	0.004	1.366	1.369
2.5	1.287*	0.014	1.282	1.293	1.335*	0.010	1.332	1.339	1.248*	0.005	1.246	1.249
3.5	1.243*	0.015	1.238	1.249	1.286*	0.016	1.280	1.292	1.170*	0.006	1.167	1.172
5	1.161*	0.023	1.153	1.170	1.120*	0.017	1.194	1.207	1.037*	0.007	1.035	1.040
7.5	0.968*	0.013	0.963	0.973	1.015*	0.019	1.008	1.022	0.819*	0.006	0.816	0.821

* *P* < 0.05 as compared with soft tissue = 0 cm

**Table 2:** BMD of a Lumbar Spine Phantom with Various Thicknesses of Soft Tissue Equivalent Material

Soft	Hologic Fast Array (Hfa)				Hologic HD (Hhd)				iDXA			
Tissue			95%CI				95%CI				95%CI	
(cm)	mean	SD	lower	upper	mean	SD	lower	upper	mean	SD	lower	upper
0	1.006	0.004	1.004	1.007	1.002	0.004	1.001	1.003	1.139	0.001	1.138	1.139
1	1.009	0.003	1.008	1.010	1.005*	0.004	1.004	1.007	1.136*	0.001	1.136	1.137
2.5	1.009	0.003	1.008	1.010	1.005*	0.003	1.004	1.006	1.132*	0.001	1.131	1.132
3.5	1.008	0.004	1.007	1.010	1.005*	0.002	1.004	1.006	1.129*	0.001	1.129	1.129
5	1.011*	0.004	1.009	1.012	1.008*	0.004	1.006	1.010	1.127*	0.001	1.127	1.128
7.5	1.019*	0.008	1.016	1.022	1.009*	0.005	1.007	1.011	1.126*	0.001	1.125	1.126

* *P* < 0.05 as compared with soft tissue = 0 cm

**Table 3:** Least significant change (LSC) of TBS and BMD

Mode	Hfa	Hhd	iDXA
TBS LSC (%)	3.62	1.74	0.97
TBS Reproducibility (%)	96.4	98.3	99.0
BMD LSC (%)	1.10	1.11	0.24
BMD Reproducibility (%)	98.9	98.9	99.8

*Hfa* Hologic fast array; *Hhd* Hologic high definition

A one-way repeated measures ANOVA was run on a set of 30 scans at each thickness of overlying soft tissue (0, 1, 2.5, 3.5, 5, and 7.5 cm) to determine if there were differences in TBS. There were statistically significant differences in mean TBS among different thicknesses of soft tissue equivalent materials for all three scan types: F(5, 145) = 2439.74, *p* < 0.001; F(5, 145) = 3529.19, *p* < 0.001; and F(5, 145) = 45,141.74, *p* < 0.001 for Hfa, Hhd, and iDXA, respectively, with or without Greenhouse-Geisser correction for sphericity . A Tukey post-hoc test revealed that, compared with no soft tissue, TBS became statistically significantly lower when the soft tissue thickness was at least 1 cm: −0.0246 ± 0.0044, *p* < 0.001; −0.0319 ± 0.0036, *p* < 0.001; and −0.0552 ± 0.0015, *p* < 0.001 for Hfa, Hhd, and iDXA, respectively.

**Fig. 2:**
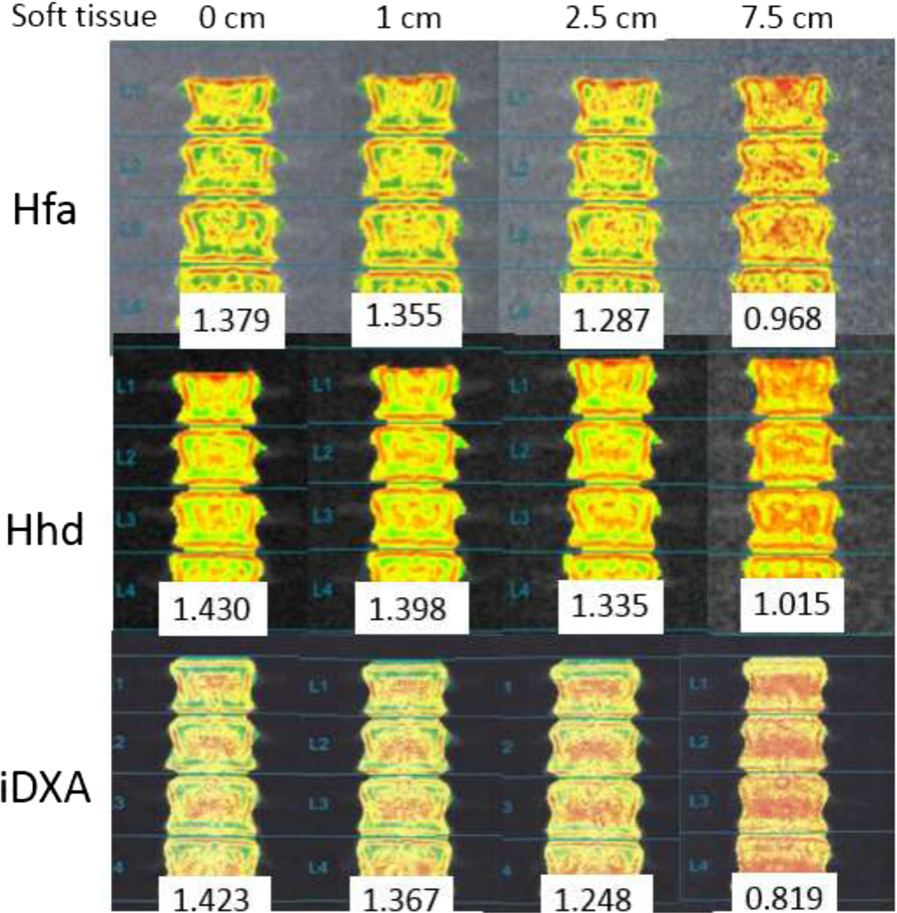
Phantom TBS images. TBS images and values of a Hologic BMD spine phantom by Hologic fast array (Hfa), Hologic high definition (Hhd), and Lunar iDXA with overlying soft tissue material of 0, 1, 2.5, and 7.5 cm

**Fig. 3:**
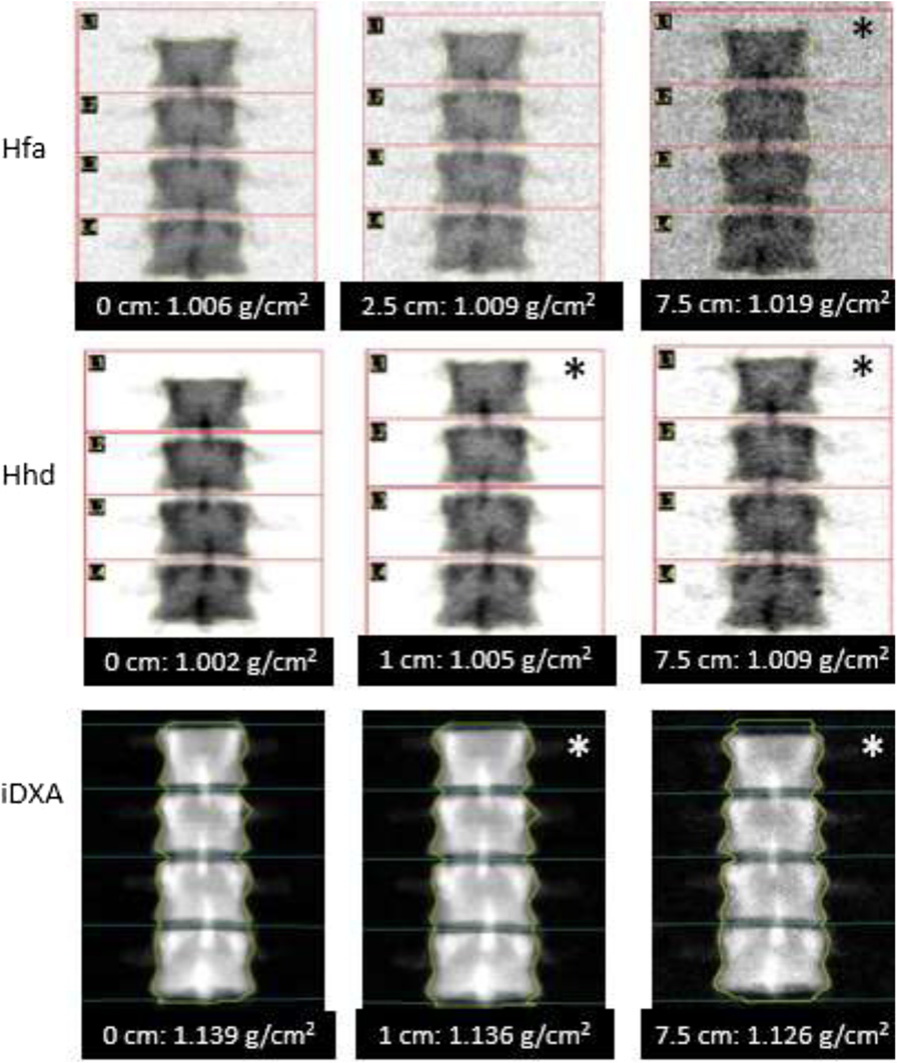
Phantom BMD images. BMD images of a Hologic BMD spine phantom by Hfa (**a**), Hhd (**b**), and iDXA (**c**) without and with overlying soft tissue equivalent materials. * Denotes the least thickness of soft tissue equivalent material that caused a statistically significant difference in BMD. Despite statistically significant changes, the absolute changes in BMD are smaller than LSC values; hence, unlikely to be a problem in BMD monitoring

**Fig. 4:**
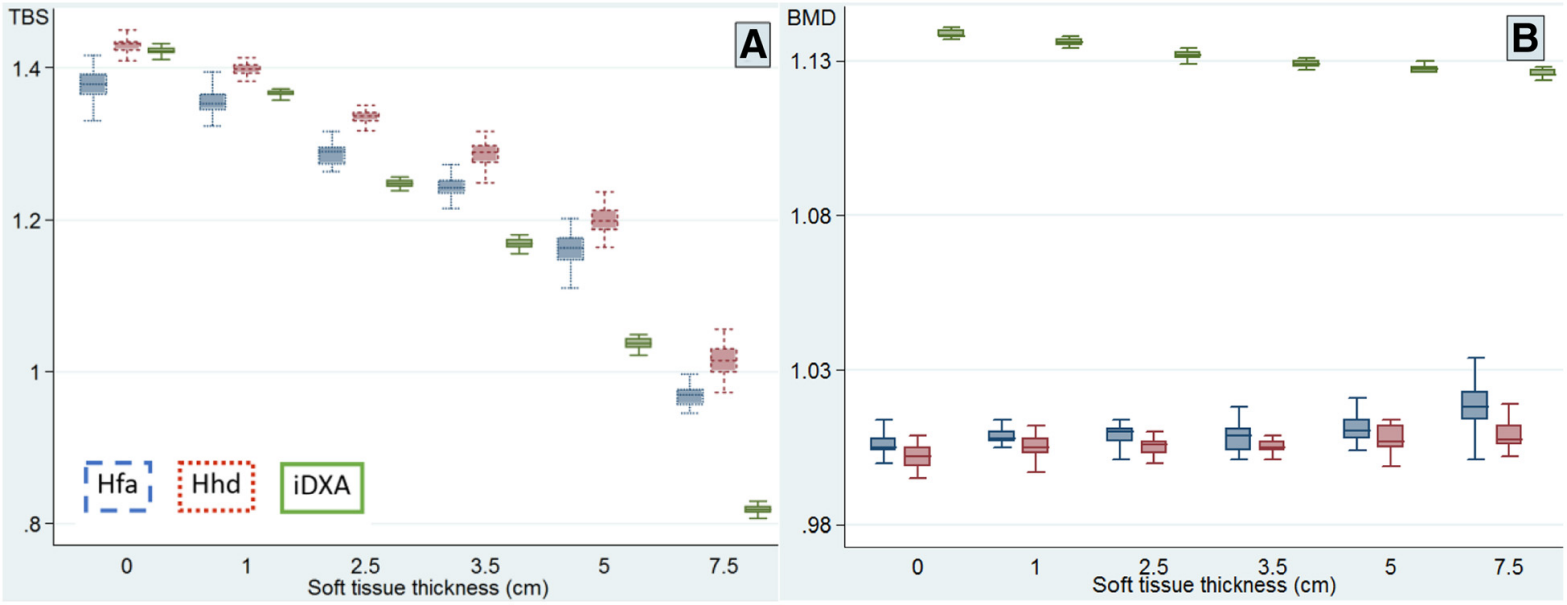
Box-and-whisker plots of TBS and BMD. Box-and-whisker plots of TBS (**a**) and BMD (**b**) with various thicknesses of overlying soft tissue equivalent materials using three scan types: Hfa (*blue dashed line*), Hhd (*red dotted line*), and iDXA (*green solid line*)

Similarly, there were statistically significant differences in mean BMD among different thicknesses of soft tissue equivalent materials for all three scan types: F(5, 145) = 27.31, *p* < 0.001; F(5, 145) = 10.47, *p* < 0.001; and F(5, 145) = 505.52, *p* < 0.001 for Hfa, Hhd, and iDXA, respectively. A Tukey post-hoc test revealed that, compared with no soft tissue, BMD became statistically significantly higher by 0.0050 ± 0.0012 g/cm^2^ (*p* = 0.001) for Hfa when the soft tissue was 5 cm thick, and by 0.0032 ± 0.0010 g/cm^2^ (*p* < 0.001) for Hhd when the soft tissue thickness was 1 cm. On the contrary, as compared with no overlying soft tissue, iDXA BMD was significantly lower, by 0.0026 ± 0.0003 g/cm^2^ (*p* < 0.001), when the soft tissue thickness was 1 cm. The effect of increased overlying soft tissue thickness on TBS and BMD could also be appreciated on scan images (Figs. 2 and 3) and is well represented on box-and-whisker plots (Fig. 4).

## Discussion

In our previous study to derive an age-adjusted TBS reference curve [9], we found a negative association between TBS and BMI. Our study on a phantom showed that increased soft tissue thickness significantly decreased TBS, regardless of the mode of acquisition or of the DXA system used, be it Hfa, Hhd, or iDXA. This finding suggests that some, if not all, of an apparent decrease in TBS with increasing weight is artefactual and does not reflect actual decreases in bone quality with increasing BMI. For any acquisition mode or DXA system, the DXA image became fuzzier with increased soft tissue thickness (Fig. 3). The soft tissue seemed to act as noise or a blurring filter. As a result, there was less variations among the image pixels; therefore, lower TBS value. This was in line with a review by Bousson et al. [14], who showed that adding noise tended to lower the TBS values.

To explore if this was also true in vivo, one of the investigators volunteered to undergo TBS measurement using Hfa mode twice without repositioning, first with and then without l-cm thick soft tissue equivalent material. Similar to the study results, TBS decreased from the baseline of 1.317 to 1.281 (2.7 %), whereas BMD increased from 0.757 to 0.767 g/cm^2^ (1.3 %). When soft tissue thickness was increased by 1 cm, the decreases in TBS were 1.74, 2.24, and 3.94 % for Hfa, Hhd, and iDXA, respectively. These decreases in TBS exceeded their phantom LSC values for Hhd and iDXA (Table 3). One may argue that in clinical practice, LSC is derived from representative patients with repositioning, and its value is probably larger and less prone to be exceeded. Indeed, the TBS LSC value derived from volunteers of 5.68 % (2.77 x 2.05 %) for Hfa [9] supports this view. Nonetheless, our study does have two clinical implications. First, for a given bone quality, subjects with thicker soft tissue (as probably seen with subjects with higher BMI) are likely to have lower TBS. Secondly, TBS could appear to be decreased in subjects who gain soft tissue thickness (such as those who gain weight) during a course of follow-up. Those who develop ascites may possibly suffer the same effect.

The finding that values of TBS displayed larger variations than BMD may be explained by their different principles. BMD is calculated by BMC/area. The BMC value is derived from the amount of radiation absorbed in each pixel, which is shown on a DXA image in a grey scale. Although each has its own precision error, the measured value is independent from one pixel to another. On the other hand, TBS is based on grey-scale variation among these pixels, a method involves comparison [14], where not only the magnitude but also the direction (increase or decrease) of change are taken into account. Hence, it is understandable that TBS exhibits larger variations.

Although to a lesser degree, there was also a statistically significant relationship between the changes in BMD and increased soft tissue thickness (Table 2 and Fig. 4). Perhaps due to algorithm difference in soft tissue handling in the calculation, the direction of change was negative for iDXA but positive for both Hfa and Hhd modes. For Hfa, the increase in BMD did not reach statistical significance until the soft tissue was 5 cm thick, causing 0.5 % increase. Even when the soft tissue thickness was 7.5 cm, the increase was only 1.3 %, which was still below our volunteer-derived LSC of 1.91 % [9]. For Hhd and iDXA, the differences in BMD were statistically significant when the soft tissue was only 1 cm thick. Nevertheless, the resulting increases in Hhd BMD of 0.3–0.7 % did not exceed its LSC at any thicknesses of soft tissue. As for iDXA, increasing the soft tissue thickness to 7.5 cm resulted in 1.14 % decrease in BMD, which was below our LSC derived from the patients of 1.19 %, although it exceeded the phantom LSC of 0.24 % (Table 3). In agreement with many previous reports [21–24], our earlier work [25] showed a strong positive association between BMI and BMD. In clinical practice, the changes in BMD as a result of overlying soft tissue in individuals are likely to be of small magnitude as compared with those as a result of BMI per se. Moreover, it may not be common for individuals to gain such weight that their soft tissue thicknesses are increased by such an amount during follow-up. Nevertheless, in comparing BMD between individuals, one should be aware that a statistically significant difference may be partly contributed by the difference in soft tissue thickness.

### Limitations

The limitations of this study are as follows. First, the thickness of the soft tissue equivalent material used could not be directly translated into the amount of weight or BMI gain; hence, recommendations in terms of weight or BMI threshold, as well as the magnitude of their effect, cannot be explicitly made. Secondly, the study was performed on one phantom only. The effect of soft tissue thickness may be more or less pronounced in individuals with lower or higher BMD and TBS values, although intuitively lower values are likely to be more affected. Finally, our study did not include acquisitions using the array mode of the Hologic system because we were only interested in comparing the fast array mode, which we use in our routine clinical practice, with the HD mode.

## Conclusions

Increased soft tissue thickness results in lower TBS value. Although BMD is also affected, it is unlikely to pose a clinical problem because the change is unlikely to exceed the patient-derived LSC.

## Abbreviations

BMD: Bone mineral density
BMI: Body mass index
DXA: Dual X-ray absorptimetry
Hfa: Hologic with fast array mode
Hhd: Hologic with high definition mode
LSC: Least significant change
ROI: Region of interest
TBS: Trabecular bone score
